# Supplementary material for: Demonstration of the potential of environmental DNA as a tool for the detection of avian species
Source: Sci Rep. 2018 Mar 14;8:4493. doi: 10.1038/s41598-018-22817-5 (PMC5851996; doi:10.1038/s41598-018-22817-5)
Supplement: Supplementary file 1 — Supplementary information [file 41598_2018_22817_MOESM1_ESM.pdf]

Supplementary Information for

Demonstration of the potential of environmental DNA as a tool for the detection of avian species

Masayuki Ushio, Koichi Murata, Tetsuya Sado, Isao Nishiumi, Masamichi Takeshita, Wataru Iwasaki, Masaki Miya

Contents:

Table S1: Critical information for MiBird environmental DNA experiment

Table S2: Number of reads that remained in data pre-processing

Table S3: Binding capacity of MiBird-U primers to Mammalia, Amphibia and Reptilia mitochondrial 12S

Table S4: Non-target sequence reads from water samples collected in the zoo

Table S1. Critical information for MiBird environmental DNA experiment

Stage	Information for	Information
Design	Inferential goal	Detection of the presence of avian species from forest pond water (tests of MiBird primers)
Water collection	Contamination precautions including negative controls	All materials were washed with 10% commercial bleach solution
	Collection volume, container material, replicates, depth	Approx. 100–200 ml water samples were filtered using φ0.45-µm Sterivex^{TM} filter cartridge (Merck Millipore). Three field negative controls were included in the zoo experiment.
	Site descriptions	Zoo survey: Yokohama Zoorasia<br>Pond survey: A pond adjucent to Natural History Museum and Institude, Chiba
Sample preservation	Method, temperature, duration	Zoo survey: Immediately taken back to lab and filtered<br>Pond survey: Immediately taken back to lab and filtered
	Filter type, filtering location	φ0.45-µm SterivexTM filter cartridge (Merck Millipore)
Extraction process	Contamination precausionts, negative controls	All materials were washed with 10% commercial bleach solution. Three distilled water samples were used as field negative controls.
	Methods including kit protocol adjustments	DNAs were extracted using Qiagen Blood & Tissue kit.
High-throughput sequencing	Library type	PCR amplicon
	Library preparation protocol	Target region was amplified by 1st PCR, and the adaptor and index sequences were combined by 2nd PCR
	Platform, read length, read pairing, expected fragment size	Sequenced by Illumina MiSeq V2 150 paired end (300 bp)<br>Expectd fragment size (with adaptor) = *ca*. 370 bp
	Primers, sequencing adaptors, sample index tags, exogenous spike-ins	MiBird primers (this study) and TruSeq Adaptors were used. Index sequences were from Hamady et al. (2008)
	Amplicon locus, target taxa, specificity and bias	12S mitochondrial DNA of avian species
	Read trimming and filtering of artefacts/chimeras	SOLEXAQA and Custam perl scripts
	Reference database	Custom (NCBI Organella Resources database)
	Taxonomic assignment method and parameters	BLAST hit, > 97% identity and < E-value 10^{-5}
	Statistical analysis and rarefaction	N.A.
	Positive and negative controls and their interpretation	Two PCR negative controls were included.
	Technical replicates and their interpretation	Three replicates in the 1st PCR were combined
	Number of raw reads and final reads	Raw reads: 1,195,286<br>Final (post-processing) reads: 1,179,808<br>Taxa assigned reads: 656,472

Table S2. Number of reads that remained in data pre-processing

Sample name	Scientific name	(0) Raw reads	(1) Tail-trimming	(2) PE assembly	(3) N removal	(4) Length filtering	(5) Primers removal	(6) Clustering and taxonomic assignment	Raw reads (0) / Final reads (6) (%)
Samples									
Steller's sea eagle	*Haliaeetus pelagicus*	47,895	47,895	47,546	47,546	47,538	47,510	**31,686**	**66.2%**
Black-tailed gull	*Larus crassirostris*	23,566	23,566	23,441	23,441	23,435	23,421	**22,359**	**94.9%**
Capercaillie	*Tetrao urogallus*	59,445	59,445	59,004	59,004	59,001	58,963	**55,585**	**93.5%**
Lady Amherst's pheasant	*Chrysolophus amherstiae*	50,773	50,773	50,383	50,383	50,376	50,346	**47,321**	**93.2%**
Ruddy shelduck	*Tadorna ferruginea*	28,484	28,484	28,287	28,287	28,280	28,262	**26,382**	**92.6%**
Temminck's tragopan	*Tragopan temminckii*	62,310	62,310	61,902	61,902	61,899	61,856	**59,392**	**95.3%**
Victoria crowned pigeon	*Goura victoria*	36,350	36,350	35,786	35,786	35,786	35,759	**26,341**	**72.5%**
Mandarin duck	*Aix galericulata*	29,373	29,373	29,142	29,142	29,135	29,110	**26,141**	**89.0%**
Humboldt penguin	*Spheniscus humboldti*	37,667	37,667	37,331	37,331	37,328	37,306	**32,632**	**86.6%**
Snowy owl	*Bubo scandiacus*	34,919	34,919	34,563	34,563	34,559	34,536	**30,212**	**86.5%**
Oriental white stork	*Ciconia boyciana*	41,972	41,972	41,666	41,666	41,661	41,626	**33,569**	**80.0%**
White-naped crane	*Grus vipio*	66,090	66,090	65,681	65,681	65,674	65,623	**62,903**	**95.2%**
Common crane	*Grus grus*	58,238	58,238	57,940	57,940	57,938	57,902	**55,851**	**95.9%**
Southern ground hornbill	*Bucorvus leadbeateri*	45,268	45,268	44,802	44,802	44,791	44,763	**42,018**	**92.8%**
Harris's hawk	*Parabuteo unicinctus*	25,697	25,697	25,463	25,463	25,460	25,446	**22,280**	**86.7%**
Emu	*Dromaius novaehollandiae*	6,992	6,992	6,746	6,746	6,746	6,741	**5,715**	**81.7%**
Field negative controls									
Field NC		28,605	28,605	28,433	28,433	28,427	28,409	**27,218**	**95.2%**
Field NC		8,735	8,735	8,681	8,681	8,680	8,675	**7,977**	**91.3%**
Field NC		44,626	44,626	44,282	44,282	44,267	44,234	**40,890**	**91.6%**
PCR negative controls									
PCR NC		120	120	21	21	21	20	**0**	
PCR NC		18	18	10	10	10	10	**0**	
Total		1,195,286	1,195,286	1,182,443	1,182,443	1,180,653	1,179,808	**656,472**	

Table S3. Binding capacity of MiBird-U primers to Mammalia, Amphibia and Reptilia mitochondrial 12S

Edit distance		0	1	2	3	4	≥ 5	Total
Biding capcacity to Mammalia mitochondrial 12S (741 species)								
Forward primer	G/T pairs not accepted	0	139	377	109	62	54	741
	G/T pairs accepted	145	25	382	146	34	9	741
Reverse primer	G/T pairs not accepted	664	58	15	3	0	1	741
	G/T pairs accepted	676	58	6	0	0	1	741
Biding capcacity to Amphibia mitochondrial 12S (197 species)								
Forward primer	G/T pairs not accepted	0	2	11	20	61	103	197
	G/T pairs accepted	18	40	45	49	17	28	197
Reverse primer	G/T pairs not accepted	120	28	37	12	0	0	197
	G/T pairs accepted	132	62	3	0	0	0	197
Biding capcacity to serpentea, sauria, testudines, and crocodylia mitochondrial 12S (245 species)								
Forward primer	G/T pairs not accepted	0	3	16	44	76	106	245
	G/T pairs accepted	20	29	67	52	51	26	245
Reverse primer	G/T pairs not accepted	81	3	96	20	38	7	245
	G/T pairs accepted	82	84	24	16	36	3	245

Table S4. Non-target sequence reads from water samples collected in the zoo

**Bird name living in cage**	**Scientific name**	Bird				Fish					Mammal		Non target total
		Mareca falcata	*Tadorna tadorna*	*Gallinula chloropus*	Other birds	*Gnathopogon elongatus*	*Carassius auratus*	*Pseudorasbora parva*	*Rhinogobius brunneus*	Other fish	*Homo sapiens*	Other mammals	
Steller's sea eagle	*Haliaeetus pelagicus*	0	0	0	646	109	0	227	0	508	1,621	127	3,238
Black-tailed gull	*Larus crassirostris*	0	0	0	3,108	74	86	229	0	1,580	12,845	0	17,922
Capercaillie	*Tetrao urogallus*	70	0	0	98	1,460	1,131	1,505	1,072	2,150	11,791	200	19,477
Lady Amherst's pheasant	*Chrysolophus amherstiae*	39	0	0	194	1,450	619	617	399	973	3,811	28	8,130
Ruddy shelduck	*Tadorna ferruginea*	32	3,552	0	79	77	0	85	82	277	8,255	59	12,498
Temminck's tragopan	*Tragopan temminckii*	0	0	0	491	63	135	0	0	100	942	213	1,944
Victoria crowned pigeon	*Goura victoria*	739	0	0	1,147	1,124	1,399	1,037	452	630	6,580	0	13,108
Mandarin duck	*Aix galericulata*	42	133	13	66	2,768	1,620	1,429	1,133	1,222	3,846	0	12,272
Humboldt penguin	*Spheniscus humboldti*	1,621	682	177	555	4,198	2,076	1,957	2,385	5,249	9,128	63	28,091
Snowy owl	*Bubo scandiacus*	1,506	1,394	567	1,097	2,263	2,019	1,597	955	4,559	12,378	231	28,566
Oriental white stork	*Ciconia boyciana*	2,654	1,419	1,328	1,279	200	762	348	425	5,577	8,697	126	22,815
White-naped crane	*Grus vipio*	62	236	0	80	122	110	111	62	452	1,113	42	2,390
Common crane	*Grus grus*	126	79	54	84	61	164	32	48	736	1,170	32	2,586
Southern ground hornbill	*Bucorvus leadbeateri*	472	362	110	904	39	39	0	51	1,309	1,451	163	4,900
Harris's hawk	*Parabuteo unicinctus*	1,361	1,302	1,055	1,448	243	154	24	121	7,061	7,283	286	20,338
Emu	*Dromaius novaehollandiae*	0	44	20	0	686	464	199	209	372	1,418	0	3,412
Field NC		0	0	0	0	160	0	0	0	0	27,058	0	27,218
Field NC		0	0	0	0	85	0	0	0	2,547	5,345	0	7,977
Field NC		709	50	209	70	1,798	1,290	1,482	1,214	2,125	31,900	43	40,890
PCR NC		0	0	0	0	0	0	0	0	0	0	0	0
PCR NC		0	0	0	0	0	0	0	0	0	0	0	0
Total sequence		9,433	9,253	3,533	11,346	16,980	12,068	10,879	8,608	37,427	156,632	1,613	277,772
